# Integrated Analysis of DNA Methylation and Gene Expression in Porcine Placental Development

**DOI:** 10.3390/ijms24065169

**Published:** 2023-03-08

**Authors:** Baohua Tan, Chen Zhou, Xupeng Zang, Xinming Zhao, Liyao Xiao, Jiekang Zeng, Linjun Hong, Zhenfang Wu, Ting Gu

**Affiliations:** 1 National Engineering Research Center for Breeding Swine Industry, College of Animal Science, South China Agricultural University, Guangzhou 510642, China; 2 Guangdong Provincial Key Laboratory of Agro-Animal Genomics and Molecular Breeding, College of Animal Science, South China Agricultural University, Guangzhou 510642, China

**Keywords:** DNA methylation, pig, placental development, transcriptome

## Abstract

Proper placental development is crucial for the conceptus to grow and survive, because the placenta is responsible for transporting nutrients and oxygen from the pregnant female to the developing fetus. However, the processes of placental morphogenesis and fold formation remain to be fully elucidated. In this study, we used whole-genome bisulfite sequencing and RNA sequencing to produce a global map of DNA methylation and gene expression changes in placentas from Tibetan pig fetuses 21, 28, and 35 days post-coitus. Substantial changes in morphology and histological structures at the uterine–placental interface were revealed via hematoxylin–eosin staining. Transcriptome analysis identified 3959 differentially expressed genes (DEGs) and revealed the key transcriptional properties in three stages. The DNA methylation level in the gene promoter was negatively correlated with gene expression. We identified a set of differentially methylated regions associated with placental developmental genes and transcription factors. The decrease in DNA methylation level in the promoter was associated with the transcriptional activation of 699 DEGs that were functionally enriched in cell adhesion and migration, extracellular matrix remodeling, and angiogenesis. Our analysis provides a valuable resource for understanding the mechanisms of DNA methylation in placental development. The methylation status of different genomic regions plays a key role in establishing transcriptional patterns from placental morphogenesis to fold formation.

## 1. Introduction

The placenta is an organ formed during pregnancy that supports the intrauterine regulation of fetal growth. It plays critical roles in the exchange of nutrients, waste, and gas; maternal–fetal immune tolerance; and metabolic and endocrine functions via the mother’s blood supply during fetal development [1,2]. Normal placental function is essential to the growth of a healthy fetus. Placental inflammation, often presenting as acute chorioamnionitis, can lead to placenta-related preterm birth [3]. Placental insufficiency may cause pre-eclampsia or intrauterine growth restriction because of the deficient remodeling of the uterine spiral arteries supplying the placenta [4,5]. Dysfunctional placental development is the main cause of fetal loss and reduced piglet birth weight, which are the primary factors associated with reproductive performance [6,7,8]. Unlike human placenta, pigs have a non-invasive, diffuse, epitheliochorial type of placenta, in which the porcine uterine epithelium remains intact instead of invading the uterine wall throughout gestation [1,2,9,10]. At around 15–20 gestation days in the pig, the trophoblast epithelial layer attaches to endometrial epithelial cells to form the epithelial bilayer [11]. Thereafter, the microscopic fold structure of the adhered trophoblast–endometrial epithelial bilayer is formed to expand the maternal–fetal exchange surface area, which reach a steady state at around 50 days of gestation [12]. Then, the folded bilayer increases in complexity and size as gestation progresses to further increase the maternal–fetal exchange surface area, leading to increased efficiency of nutrient absorption, thermal regulation, waste disposal, and gas exchange to accommodate fetal growth [13]. Thus, the development of a folded bilayer is crucial for placental function and efficiency, but the molecular mechanism of this process needs to be further elucidated. 

A previous study revealed the morphological structures of porcine placenta after 26, 50, and 95 days of gestation and found that heparinase (HPSE) may contribute to the development of the folded bilayer in pigs [13]. Many genes have been reported to participate in the proliferation and migration of trophoblast cells [14,15,16,17]. Epigenetic mechanisms, including DNA methylation, histone modifications, and ncRNAs, are involved in the regulation of gene expression and organism development [18,19]. The miRNA and lncRNA expression patterns and genome-wide profiles of histone modifications H3K27ac and H3K4me3 in porcine placental development during the establishment and expanding stages of the fold structure have been revealed [11,12,20,21]. DNA methylation is a critical epigenetic modification involving the transfer of a methyl group onto the fifth carbon of cytosine to form 5-methylcytosine [22]. It usually occurs in the context of cytosine-phospho-guanine (CpG) dinucleotides and is catalyzed by a family of DNA methyltransferases (DNMTs). DNMT3A and DNMT3B can establish new methylation patterns on unmodified DNA, whereas DNMT1 can copy the DNA methylation pattern from the parental DNA strand onto the newly synthesized strand during DNA replication [23]. DNA methylation acts as a barrier to gene transcription by preventing the binding of transcription factors (TFs) to DNA [24], which is crucial for the regulation of neuronal differentiation and maturation [25], tumorigenesis [26], and aging [27]. Recent studies have also pointed out the important role of DNA methylation during placental development. DNA methylation induced by embryonic signals could contribute to regulating endometrial gene expression during pregnancy establishment [28]. Abnormal DNA methylation may change placental formation, which may lead to adverse pregnancy outcomes [4]. The proliferation of trophoblast cells can be inhibited and the embryo implantation rate can be remarkably decreased by administering 5-Aza-CdR (DNA methylase inhibitor) in pregnant mice [29]. DNA demethylase (TET1/2) can affect fetal development by regulating the integrity of trophoblast cells and the intracellular replication cycle [29]. Several high-throughput analyses have been performed to analyze the DNA methylation profiles of specific placental tissues. Hwang et al. (2017) collected the placentas of Berkshire pig with different litter sizes to explore the function of DNA methylation [22]. Luo et al. (2021) conducted a genome-wide analysis of DNA methylation and gene expression profiles in placenta samples from nine Dazu black goats after 20, 25, and 30 days of pregnancy [30]. However, DNA methylation changes in porcine placentas at different developmental periods are still not well established.

The Tibetan pig is a rough-feeding-tolerant and hyper-prolific pig breed with an average of seven piglets born alive per litter and 114 days of gestation [31]. In this study, we performed RNA-seq and whole-genome bisulfite sequencing to provide the first integrated analysis of DNA methylation and gene expression profiles in the developing placentas of Tibetan pigs 21, 28, and 35 days post-coitus (hereafter denoted as p21, p28, and p35, respectively). We aimed to reveal the regulatory mechanisms of DNA methylation involved in placental development, especially the formation and expansion of the fold structure. The dynamic changes in gene expression in these three stages and the key pathways associated with placental development were identified. We revealed the distribution characteristics of DNA methylation and highlighted its critical role in regulating gene expression during placental development. This study can provide a basis for expanding current understanding of the regulatory mechanisms of placental development.

## 2. Results

### 2.1. Morphology and Gene Expression changes during porcine Placental Development 

**Figure 1:**
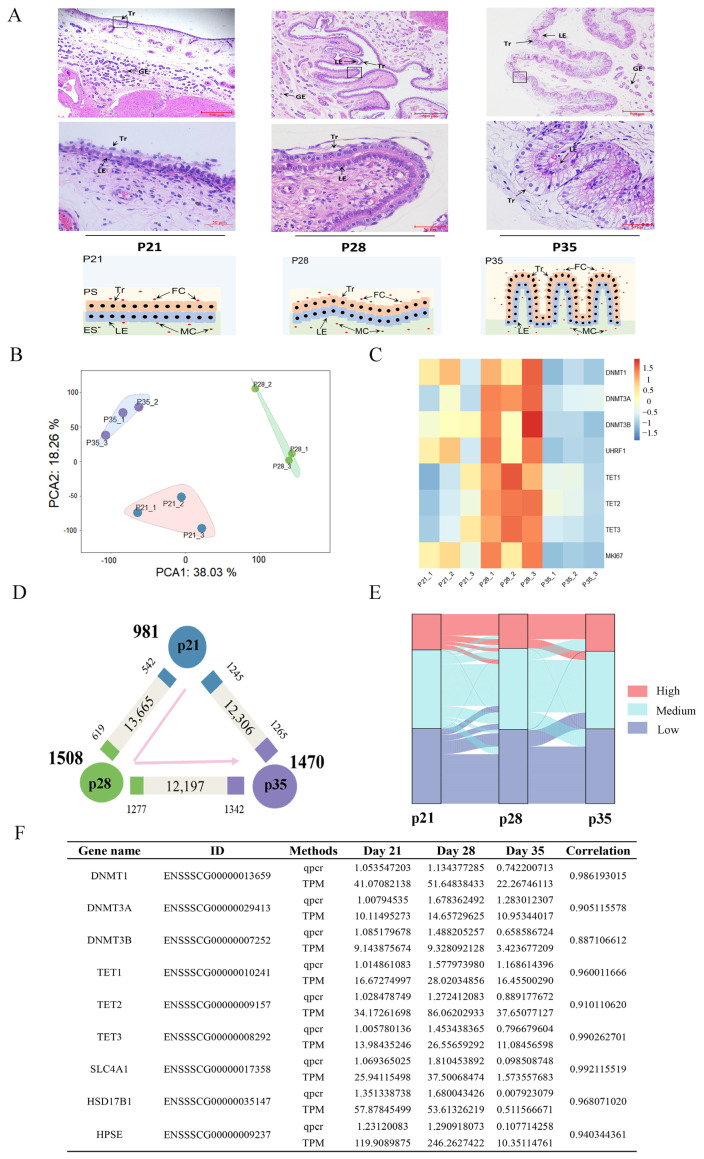
Histological structure and gene expression change during porcine placental development. (**A**) H&E staining and morphological changes of placenta on different gestation days, with the placenta seen at 10× magnification (upper panel) and in further detail at 40× magnification (middle panels). Schematic drawing of the placental structure as gestation advances in Tibetan pigs (lower panels). GE—glandular epithelium; LE—endometrial luminal epithelial; Tr—trophoblasts; FC—fetal capillaries; MC—maternal capillaries; PS—placental stroma; ES—endometrial stroma. Scale bar = 500 μm (upper) and 50 μm (middle). (**B**) The results of PCA on gene expression data. (**C**) Heatmap showing the expression levels of DNA methyltransferases and demethylases during placental development based on RNA-seq. (**D**) Gene expression changes. The numbers of genes that are upregulated, downregulated (both marked by the corresponding colors), or unchanged (gray) at each stage compared with their neighboring stage are indicated. The numbers of genes that are expressed at the highest level in each stage are indicated in bold next to each stage. (**E**) An alluvial diagram of DEGs. High (TPM ≥ upper quartile); medium (lower quartile < TPM < upper quartile), low (TPM ≤ lower quartile). (**F**) Validation of RNA-seq results using quantitative RT-PCR.

Porcine placental trophoblast cells are columnar epithelial cells, and the morphology of the epithelium is closely related to its function. The implantation of pig embryos is completed after about 19 days of gestation, and the placenta formed after implantation is epithelial chorionic placenta [2,9,32]. The morphological characteristics of porcine placental tissues on p21, p28, and p35 were microscopically evaluated via hematoxylin–eosin (H&E) staining (Figure 1A). The stained section revealed that the trophoblast cells began to adhere in the uterine luminal epithelium to form the trophoblast–endometrial epithelial bilayer on day 21 of pregnancy. The folded structure of the epithelial bilayer of the placenta began to form, but only a few irregular microscopic folds appeared on p28. On p35, the adhered trophoblast–endometrial epithelial bilayer formed regular microscopic folds, and the folds deepened further and became wider and more complex than those on p28. 

A total of 859,742,968 clean reads were generated from nine porcine placenta samples obtained on p21, p28, and p35 with three replicates each, and more than 81% of the clean reads were mapped to the pig reference genome (Supplementary Table S1). The closed clustering of biological replicates in the principal component analysis (PCA) plots indicate highly reproducible results (Figure 1B). DNA methylation is dynamically regulated by DNA methyltransferases and demethylases. We examined the expression of DNA methyltransferase and demethylase genes to study the dynamic change in DNA methylation status via RNA-seq data (Figure 1C). We observed that the DNA methyltransferases and demethylases showed the highest expression on p28 and the lowest expression on p35, which coincide with the proliferation status of trophoblast cells, as evidenced by the expression change of the proliferation marker protein, Ki67. The expression of methyltransferases was higher than that of demethylases on p21, suggesting that the de novo DNA methylation was more activated. In this study, we identified 3959 differentially expressed genes (DEGs) among three stages. We explored the genes that were upregulated or downregulated among the successive stages and identified those expressed at the highest level in p21 (981, namely, “the p21 genes”), p28 (1508, namely, “the p28 genes”), or p35 (1470, namely, “the p35 genes”; Figure 1D). We generated an alluvial diagram to reflect the degree of differences in expression. We found 20 DEGs with dramatic changes in expression, such as *SLC4A1*, which is involved in transmembrane transport, and *HSD17B1*, which is involved in the estrogen biosynthetic process (Figure 1E). The relative expression levels of genes from RT-qPCR were highly correlated with those obtained from transcriptome sequencing (Figure 1F). Collectively, we revealed the dynamic changes in gene expression across the three stages during placental development.

### 2.2. Functional Enrichment Analysis of Differentially Expressed Genes 

**Figure 2:**
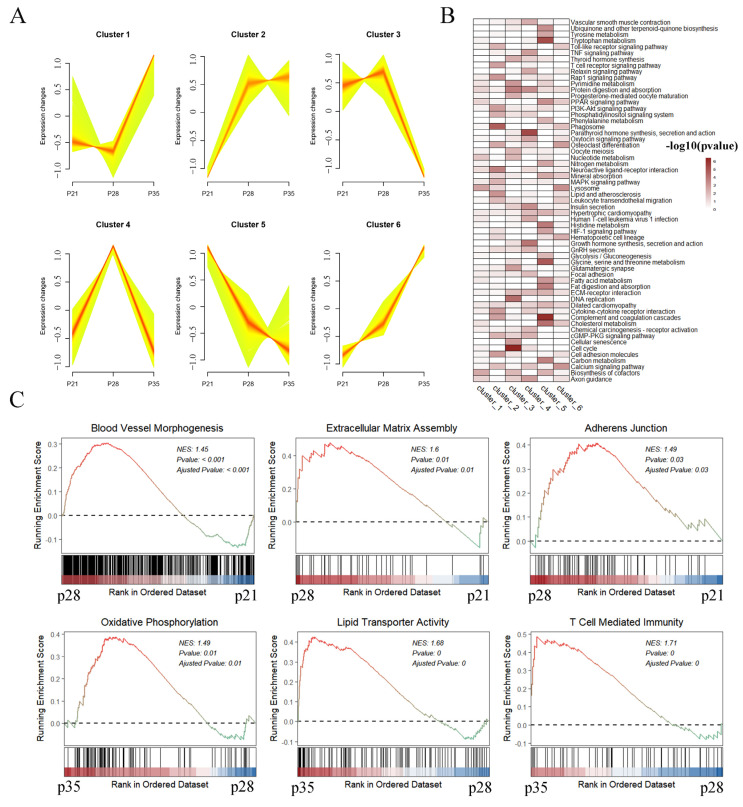
Functional enrichment analyses of differentially expressed genes. (**A**) The clustering analysis of transcriptome dataset across 3 time points. (**B**) The KEGG pathway enrichment analysis in each cluster and a summary of these pathways depicted using a heatmap. (**C**) GSEA plot of genes involved in various processes on different gestation days.

We performed soft clustering of the 3959 DEGs identified in the three development stages to better identify groups of co-regulated genes (Supplementary Table S2). The DEGs were divided into six clusters according to the trend characteristics of gene expression (Figure 2A). Then, we searched for enriched Kyoto Encyclopedia of Genes and Genome (KEGG) pathways (*p* < 0.05) within each cluster and summarized these pathways via a heatmap (Figure 2B, Supplementary Table S3). We found that metabolism-related pathways, such as tryptophan metabolism, PPAR signaling pathway, and cholesterol metabolism, were more enriched in Cluster 5 (genes with particularly high expression in p21). The expression levels of genes in Cluster 3 (genes upregulated on p21 and p28, and then, downregulated on p35) were enriched in the pathways of cell cycle and DNA replication, reflecting the dynamic status of cell proliferation activity across placental development. We also found that cytokine–cytokine receptor interaction, cell adhesion, the T cell receptor signaling pathway, and the Rap1 signaling pathway were remarkably enriched in Cluster 2 (genes with high expression in p28 and p35). Furthermore, we performed a gene set enrichment analysis of porcine transcriptome to focus on several important biological processes related to placental development. Our results showed that blood vessel morphogenesis, cell adhesion, and extracellular matrix (ECM) assembly were more activated on p28 than on p21 (Figure 2C). A comparison of the transcriptome profiles from p35 to p21 revealed that the genes with functions in lipid transporter activity, oxidative phosphorylation, and T cell-mediated immunity were highly expressed in p35 (Figure 2C). In summary, we used various functional enrichment analyses to reveal the dynamic changes in transcriptome characteristics during placental development.

### 2.3. Characteristics of DNA Methylome during Placental Development

**Figure 3:**
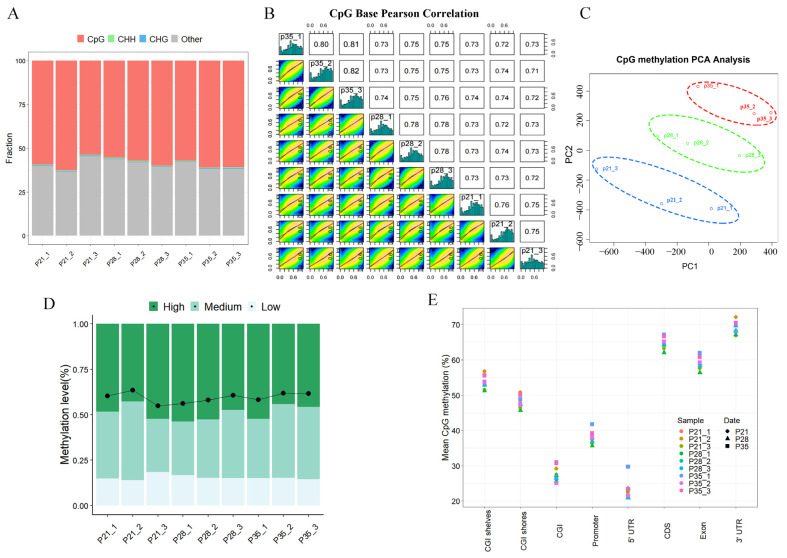
Dynamic changes in DNA methylation occur in placental development. (**A**) The distribution of methylated cytosine sites in different sequence contexts. (**B**) Scatter plots of % methylation values for each pair of samples on different gestation days. Numbers in upper right corner denote pair-wise Pearson’s correlation scores. Histograms show CpG methylation level of each sample from 0% to 100% distributed across 20 bins of 5% intervals. The red line and green line represent linear regression and loess fit, respectively, to model the relationships of differential CpG methylation sites between compared individual pairs. (**C**) Principal component analysis (PCA) of DNA methylation in each sample. Only the first and the second component are visualized. (**D**) Fraction of total CpGs with low (≤25%), medium (>25% and <75%), and high (≥75%) methylation levels in placental tissues. The ^m^CG/CG ratio, from left to right: 0.60343, 0.63564, 0.54975, 0.56215, 0.58172, 0.60793, 0.58236, 0.61802, and 0.61703. (**E**) Mean methylation level of CpGs overlapping different genomic features. CDS—coding sequence; UTR—untranslated regions.

Next, we analyzed the characteristics of DNA methylome during placental development. We generated 2.12 billion paired-end reads (150 bp × 2) covering 357 Gb of the sequence for WGBS (Supplementary Table S1). The average mapping rate of clean WGBS reads to the reference genome (Sscrofa 11.1) was 77.10% (range 74.30−78.70%). The majority of methylated cytosine sites occurred in a CpG sequence context (approximately 60%); thus, we only focused on methylated CpG sites (CpGs) in the subsequent analysis (Figure 3A). The global methylation profiles showed a high positive correlation among placental tissues on p21, p28, and p35 ranging from 0.71 to 0.82, and the methylation levels in most samples showed bimodal distribution, with peaks at low (<10%) and high (>50%) DNA methylation (Figure 3B). The PCA results revealed clear separation of the replicates at different stages, and three biological replicates at each stage were clustered (Figure 3C). The genome-wide CpG methylation levels were approximately 60% across different samples, and most of the CpG sites were heavily methylated (Figure 3D). Unmethylated CpGs were found mostly in CpG islands (CGIs), the gene promoter, and 5′ untranslated regions (UTR), whereas CpGs in the gene body, 3′ UTR, CGI shelves, and CGI shore were heavily methylated, as reported in a previous study (Figure 3E) [33]. Collectively, we revealed the distribution of methylated cytosine sites and the methylation level of CpGs in different genomic features. 

### 2.4. Dynamic Changes in DNA Methylation Occur in Placental Development

**Figure 4:**
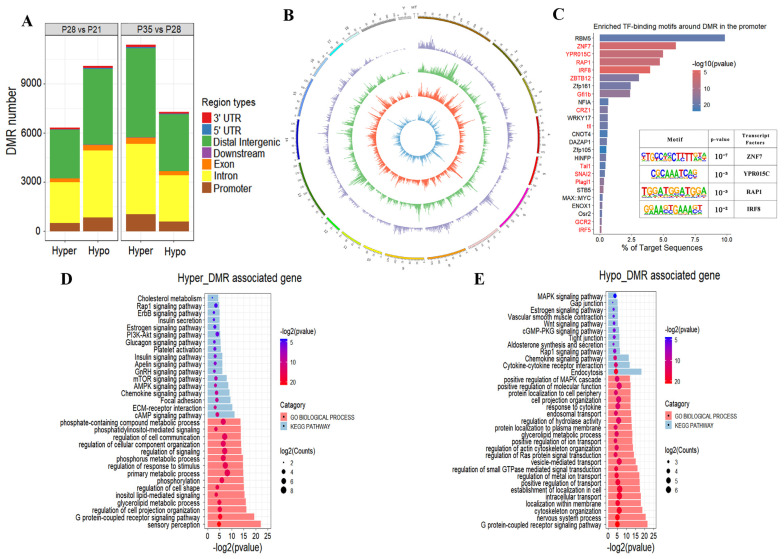
Dynamic changes in DNA methylation occur in placental development. (**A**) The distribution of DMRs on different genomic features. (**B**) Circos visualization of data corresponding to the chromosomal locations. From outermost ring to innermost ring: (1) Hyper-DMRs on p28 compared to p21. (2) Hypo-DMRs on p28 compared to p21. (3) Hyper-DMRs on p35 compared to p28. (4) Hyper-DMRs on p35 compared to p28. (**C**) Enriched TF-binding motifs around DMR in the promoter. (**D**,**E**) GO and KEGG analysis results of the genes with DMRs in promoter.

We identified differentially methylated regions (DMRs) and determined the functional implication of DMRs in placental development to explore the dynamic changes in and potential function of DNA methylation (Supplementary Table S4). Comparison of placental tissues on p28 to those on p21 revealed 6338 hypermethylated DMRs (referred to as hyper-DMRs) and 10,101 hypomethylated DMRs (referred to as hypo-DMRs; Figure 4A). We found 11,400 hyper-DMRs and 7305 hypo-DMRs from p28 to p35 (Figure 4A). As a result, we found that about 9% of DMRs occurred in the gene promoter and that the DMRs were mainly located in the intron and distal intergenic region of each sample. We visualized the DMR distribution in the genome using a Circos plot and observed that DMRs occurred on all chromosomes without obvious chromosome preference (Figure 4B). Subsequently, we combined all the DMRs identified from the comparison of p21 to p28 and p28 to p35, and focused on the region that was annotated in the gene promoter. We found that promoter-DMRs were remarkably enriched in the binding motifs of placenta-related TFs, such as ZNF7, YPR015C, and RAP1 (Figure 4C). KEGG and gene ontology (GO) function category analyses revealed that the genes with hyper-DMRs in the promoter were enriched in cholesterol metabolism, the mTOR signaling pathway, ECM–receptor interaction, and cell adhesion (Figure 4D). The genes with hypo-DMRs in the promoter were enriched in the estrogen signaling pathway, the MAPK signaling pathway, intracellular transport, and the cytokine–cytokine receptor interaction (Figure 4E).

### 2.5. Association between DNA Methylation and Gene Expression during Placental Development

**Figure 5:**
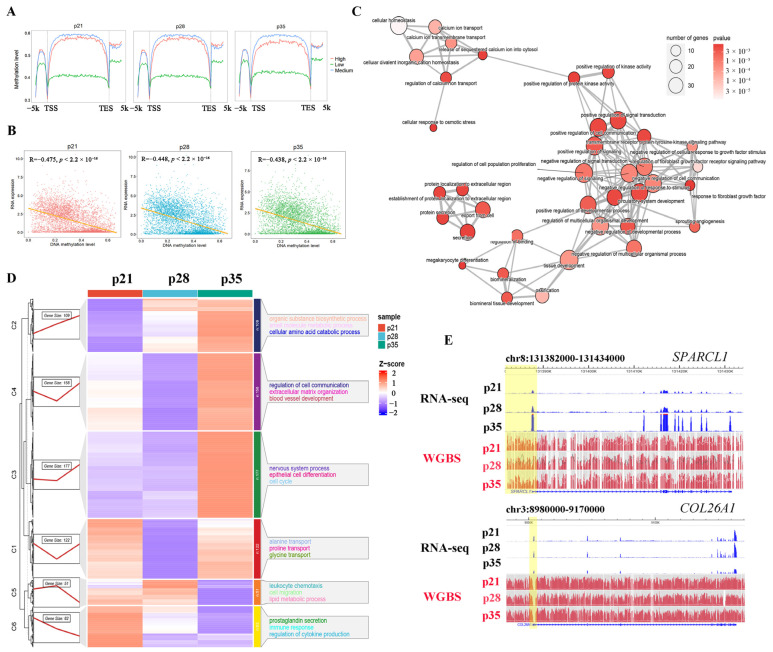
Association between DNA methylation and gene expression during placental development. (**A**) Average DNA methylation levels across and around gene bodies of genes with different expression levels at these stages. Genes were classified into three groups according to their expression level (high expression, medium expression, and low expression) via RNA-seq. (**B**) The distribution of correlation coefficients between DNA methylation (promoter) and the expression level of corresponding genes in placental development. (**C**) The GO enrichment analysis of DM-DEGs. (**D**) The clustering analysis of DM-DEGs and expression pattern across three stages. (**E**) WashU Epigenome Browser snapshot of the dynamic DNA methylation and expression patterns in the *SPARCL1* and *COL26A1* genes from p21 to p35. Gene promoter region (TSS ± 3 kb) is colored in yellow.

DNA methylation is well-known epigenetics factor that influences gene transcriptional activity [23]. We divided all the expressed genes into three groups according to their expression level (high, medium, and low) at each stage to investigate the relationship between DNA methylation and gene expression during placental development. The enrichment profile results show that the gene expression was negatively correlated with methylation levels around the transcription start site (Figure 5A). Moreover, we calculated the Pearson correlation coefficients between the DNA methylation levels and the gene expression levels of the corresponding genes across the three developmental stages. A total of 4774 genes whose DNA methylation levels on the promoter were negatively correlated with their expression levels at the whole-genome level (R<−0.475) were identified and denoted as promoter-negative genes (PNGs) (Figure 5B). We overlapped all the PNGs with the 3959 DEGs to identify the DEGs regulated by DNA methylation. Consequently, 699 DEGs overlapping PNGs were identified and denoted as DM-DEGs (Supplementary Table S5). GO analysis showed that the DM-DEGs were enriched in processes related to placental development, including extracellular structure organization, signal transduction, cell communication, angiogenesis, and transmembrane transport (Figure 5C). Hierarchical clustering based on the expression across the three stages suggested that the 699 DM-DEGs could be grouped into six clusters (C1–C6, Figure 5D). Interestingly, we found that the majority of the DM-DEGs were enriched in C3 and C4, and were significantly enriched in processes related to blood vessel development, ECM organization, and the cell cycle (Figure 5D); moreover, most of the genes with the highest expression in p21 were closely associated with amino transport, cytokine production, and immune response. The DNA methylation levels and expression patterns of two placenta-related DM-DEGs, namely, *SPARCL1* and *COL26A1*, were shown using the WashU Epigenome Browser (Figure 5E). These results suggest that DNA methylation could regulate placental development by affecting gene expression.

## 3. Discussion

The placenta is a temporary organ that connects the developing fetus to the uterine wall through the umbilical cord to allow for nutrient absorption, thermal regulation, waste disposal, and gas exchange [1,2,34,35]. The fetal demand for nutrient uptake increases rapidly with the advancement of gestation; thus, the placenta undergoes remodeling to increase placental efficiency by: (1) developing placental folds to expand the maternal–fetal exchange surface area and (2) increasing capillary density and permeability [11]. In this study, we revealed the microstructural changes, provided the dynamic and global-scale distributions of the DNA methylome profile, and analyzed the corresponding transcriptome characteristics at different stages of placental development. 

The H&E staining results showed dynamic changes in the placental structure across p21, p28, and p35. Embryo attachment is completed on day 18 in pigs, and then, the placenta begins to form [36]. Our study showed that trophoblast thickness constantly increased from p21 to p35. The regular fold structure of the placenta was not yet formed on p28 and p35. These findings were consistent with a previous study showing that the regular fold structure of the placenta is not yet formed after 26 days of gestation but forms after 50 days of gestation [12]. We identified the DEGs in porcine fetal placentas during the establishment and expanding stages of placental fold development. Gene functional enrichment analysis showed that the DEGs were enriched in blood vessel morphogenesis, cell adhesion and ECM assembly, lipid transporter activity, oxidative phosphorylation, and T cell-mediated immunity. Integrins are dominant glycoproteins in many cell adhesion cascades [37]. They can promote cell–cell and cell–ECM adhesion and control intracellular signaling events [38,39]. A previous study pointed out the important role of *ITGB3*. Blocked mRNA translation for trophectoderm-expressed *ITGB3* can impair the growth of the ovine conceptus and vasculature within the allantois, and the loss of ITGB3 protein in the conceptus was validated via immunofluorescence analysis [40]. In this study, we found that *ITGB4*, *ITGB6*, *ITGB7*, and *ITGB8* were differentially expressed in the placenta, in addition to *ITGB3*, suggesting the potential regulation of these integrins during placental development.

Growing evidence has shown that DNA methylation can impact gene expression by altering chromatin accessibility, which eventually leads to the silencing of gene expression. Interestingly, DNA methylation in different genomic regions may exert different influences on gene activities based on the underlying genetic sequence [23]. Whether DNA methylation of the gene body contributes to gene regulation is still unclear, but it is associated with gene activation or repression in dividing cells [41,42]. However, DNA methylation at the promoter has long been recognized, with a correlation between DNA methylation and gene silencing that increases with the density of CpG dinucleotides [43,44]. In this study, the DNA expression level in the gene body did not show significant correlation with gene expression. Expectedly, we found that gene expression exhibited a remarkably negative correlation with DNA methylation in the corresponding promoter at the global level, suggesting the important role of DNA methylation in regulating gene transcription during placental development. Our results suggest that DMRs widely exist in placental tissues and that DMR-annotated genes are closely associated with placental development. We further explored whether TF binding is one of the causes of DNA methylation change. TF enrichment analysis in the DMRs indicated that some reported placenta-related TFs, such as *RAP1*, were enriched in the DMRs. RAP1 is a small GTPase belonging to the Ras family of GTPases that regulates trophoblast cell proliferation, adhesion, and fusion after activation by several Rap1-related guanine nucleotide exchange factors and Ras-related guanyl releasing proteins [45,46,47]. Our results indicated that *RAP1* might mediate the regulation of DNA methylation level in some genes associated with placental development, although further investigation is required to elucidate details of the mechanisms.

The development of placental folds depends on trophoblast migration and proliferation, which are required for growth factors and ECM-degrading enzymes to participate in ECM breakdown and the promotion of trophoblast–endometrial epithelial cell proliferation [48,49]. *HPSE* is the only endogenous endoglycosidase that can degrade heparan sulfate proteoglycans (the main components of ECM) and participate in ECM remodeling, cell migration, and growth [48,50]. We found that *HPSE* is upregulated from p21 and p28 but was barely detected on p35. One possible explanation for the expression change of HPSE is that HPSE might play a role in early placentation by initiating the breakdown of HSPGs in the ECM of the maternal–fetal interface, to facilitate the attachment, migration, and initiation of folding in the epithelial bilayer. The adhered trophoblast–endometrial epithelial bilayer formed regular microscopic folds on p35 as a relative steady-state stage of development, which coincided with the downregulation of HPSE. These results are in agreement with our previous study wherein *HPSE* was highly expressed after 26 days of gestation but was barely detected after 50 days of gestation [12]. We firstly found that the gene transcription of *HPSE* might be regulated by DNA methylation, because the change in DNA methylation in the promoter of HPSE showed a highly negative correlation with gene expression (*R* = −0.99984). Secreted protein acidic and rich in cysteine-like 1 (*SPARCL1*) is an extracellular matrix protein that can impede trophoblast migration and invasion by downregulating ERK phosphorylation and AP-1 production and altering EMT-related molecule expression in HTR8/SVneo cells and JAR cells [51]. We found that *SPARCL1* expression constantly increased with the promotion of DNA methylation levels in the promoter. During fetal development, the placenta undergoes high levels of vasculogenesis and angiogenesis. Suppressing placental angiogenesis can reduce blood flow to the placenta, leading to pregnancy syndrome and, ultimately, fetal growth failure [52]. Our results showed that the gene transcription of some genes involved in blood vessel development and the apelin signaling pathway were regulated by DNA methylation during placental development, including *EDN2*, *APLN*, and *ALDH2*. Utero-placental angiogenesis and blood flow are controlled by numerous vasodilator and vasoconstrictor systems, such as endothelins (EDNs) and the renin angiotensin system [53]. *EDN2* is one of the ENDs and exhibits strong vasoconstricting activity. *EDN2*mRNA is localized to the endometrial luminal epithelial cells and glandular epithelial cells at pre-implantation [53]. Our results showed that *EDN2* had the highest expression in p35, and that gene transcription might be controlled by DNA methylation. Collectively, these findings indicate that DNA methylation might play an important role in placental development (particularly ECM remodeling and angiogenesis) by properly controlling transcription programming. 

## 4. Conclusions

Our study provides the transcriptome-wide landscape of gene expression change and of the DNA methylome at different stages in the development of placental tissues. We linked the regulation of DNA in gene transcription to the differences in the phenotypes in developing placenta. These results could provide novel clues to clarifying the underlying regulatory mechanisms of fold structure establishment and expansion during placental development.

## 5. Materials and Methods

### 5.1. Sample Collection

Nine placenta samples were collected from nine third-parity Tibetan sows (three different sows per gestation day). All Tibetan sows were half-siblings with similar ages and genetic backgrounds. The sows were housed and managed in the same pen. Gilts were mated naturally with the same boar after estrus. Gilts were euthanized on gestation days 21, 28, and 35, and their uteruses were removed immediately. Placenta samples were excised from the maternal side of the placenta, 2 cm from the umbilical cord insertion site and free of maternal decidua corresponding to the chorioallantoic placenta. The corresponding conceptuses of the placenta were healthy and without degeneration. The collected placental tissues for RNA or DNA extraction were rapidly placed in liquid nitrogen, and uteroplacenta samples for morphological observation were fixed with 4% paraformaldehyde (PFA) for further processing.

### 5.2. Hematoxylin–Eosin Staining

Histomorphometry staining on paraffin sections was performed as previously described [30]. Briefly, placenta samples were fixed in 4% PFA for 24 h at room temperature. The paraffin-embedded tissues were then sectioned (4 um thick) and placed on Superfrost Plus slides (Thermo Scientific). The sections were deparaffinized and rehydrated to prepare for hematoxylin–eosin (H&E) staining. Next, the slides were soaked in hematoxylin for 15 min and rinsed with running water for H&E staining. After 2 min, all slides were dehydrated in 70%, 95%, and 100% ethanol series for 20 s each. The slides were brightened in xylene for 3 min and covered using Permount TM Mounting Medium. Images were captured using a Nikon ECLIPSE Ci microscope (Nikon, Tokyo, Japan).

### 5.3. Real-Time Quantitative PCR (qPCR)

Total RNA was extracted using TRIzol reagent (15596018, Gibco, Carlsbad, CA, USA ) according to the manufacturer’s protocol. qPCR was performed on an ABI QuantStudio 5 Flex system (Thermo Fisher Scientific, Waltham, MA, USA) using PowerUp^TM^ SYBR^TM^ Green Master Mix (Thermo Fisher Scientific, Waltham, MA, USA). For each time-point, quantitative PCR was performed on three biological replicates, each with three technical replicates. The relative gene expression level was calculated using the Ct (2^−ΔΔCt^) method. Data were normalized to β-actin expression for each sample. The primer sequences are shown in Supplementary Table S6. 

### 5.4. RNA-seq and Data Analysis

RNA-seq was performed using three biological replicates. Total RNA was isolated using TRIzol reagent (15596018, Gibco, Carlsbad, CA, USA) according to the manufacturer’s protocol. The total amounts and integrity of RNA were assessed using the RNA Nano 6000 Assay Kit of the Bioanalyzer 2100 system (Agilent Technologies, Carlsbad, CA, USA). The libraries were generated using NEBNext R UltraTM RNA Library Prep Kit for Illumina R (E7775, New England BioLabs, Ipswich, MA, USA), and then, sequenced on an Illumina NovaSeq 6000 platform at Novogene (Beijing, China). We produced 150 bp paired-end raw reads. Clean reads were obtained by trimming reads that had an adapter, reads containing ploy-N, or low-quality reads from raw data using fastp [54]. The reference genome and the latest gene annotation files were directly downloaded from the Ensembl website [55]. Hista2 was used to build the index and map the clean reads to the reference genome of the pigs (Ensembl Sscrofa 11.1.94) with default parameters [56]. The raw gene counts were quantified via featureCounts [57] and normalized to the transcripts per kilobase million (TPM) using custom scripts. Differential gene expression analysis between the two groups was performed using the R package DESeq2 [58]. Genes with adjusted *p*-values < 0.05 following a Benjamini–Hochberg test and a log2 fold change (log2FC) ≥|1| were considered differentially expressed genes. Gene ontology (GO) analyses and Kyoto Encyclopedia of Genes and Genomes (KEGG) pathway analyses were, respectively performed using PANTHER [59] and KOBAS-i [60]. Gene Set Enrichment Analyses (GSEA) were implemented and visualized using the R package clusterProfiler [61]. The expressed genes were defined as genes with TPM ≥ 0.5 in at least one sample. Soft clustering was performed to obtain the DEGs and reveal their expression profiles based on similar expression patterns using the R package Mfuzz [62].

### 5.5. Genome Bisulfite Sequencing and Data Analysis

A total amount of 100 ng genomic DNA spiked with 0.5 ng lambda DNA was fragmented via sonication to 200–300 bp using Covaris S220 (Covaris, Woburn, MA, USA). These DNA fragments were treated with bisulfite using a Scale Methyl-DNA Lib Prep Kit from Illumina, and the library was constructed by the Novogene Corporation (Beijing, China). Subsequently, 150 bp paired-end sequencing of the samples was performed on an Illumina Novaseq platform (Illumina, CA, USA). The raw read sequences produced by the Illumina pipeline in FASTQ format were pre-processed through fastp [54]. Clean reads were obtained by trimming reads that had an adapter, reads containing ploy-N, or low-quality reads from raw data using fastp [54]. Bismark software (version 0.19.0) was used to perform alignments of bisulfite-treated reads to a reference genome with default parameters [63]. Reads that aligned to the same regions of the genome were regarded as duplicated ones and were filtered. Methylated cytosines were extracted from aligned reads using the Bismark methylation extractor with the standard parameters. We focused only on CpG methylation, and only CpGs with at least five-read coverage were retained. The methylation level of each CpG site was defined as the number of methylated reads (C) divided by the total number of methylated reads (C) and unmethylated reads (T) at the same positions in the reference genome. To compute the correlation between DNA methylation and gene expression, genes were first classified into four groups according to their expression level in RNA-seq data (high, medium, or low). We analyzed CpG DNA methylation levels in gene features (5 kb upstream, gene body, and 5 kb downstream) of the three gene groups at each stage. The gene locations were divided into 20, 100, and 20 bins for upstream 5 k, gene body, and downstream 5 k, respectively. Identification of the DMR regions and principal component analysis (PCA) analysis were performed using the R package MethyKit [64]. The R package ChIPseeker was used to annotate the DMR regions and find the nearest genes around the regions [65]. The correlation between the methylation level of the promoter and the expression level of the corresponding gene was calculated using the Pearson Correlation method. Visualization of DNA methylation and expression patterns in the selected regions was performed in the WashU Epigenome Browser [66].

## Data Availability

The datasets presented in this study have been deposited in the NCBI Sequence Read Archive (SRA); these records can be accessed using the accession numbers PRJNA910318 and PRJNA917463.

## Supplementary Materials

The following supporting information can be downloaded at: https://www.mdpi.com/article/10.3390/ijms24065169/s1.
